# Choroidal Round Hyporeflectivities in Geographic Atrophy

**DOI:** 10.1371/journal.pone.0166968

**Published:** 2016-11-23

**Authors:** Eleonora Corbelli, Riccardo Sacconi, Luigi Antonio De Vitis, Adriano Carnevali, Alessandro Rabiolo, Lea Querques, Francesco Bandello, Giuseppe Querques

**Affiliations:** 1 Department of Ophthalmology, University Vita-Salute, IRCCS Ospedale San Raffaele, Milan, Italy; 2 Department of Ophthalmology, University of Verona, University hospital of Verona, Verona, Italy; 3 Department of Ophthalmology, University of “Magna Graecia”, Catanzaro, Italy; University of Florida, UNITED STATES

## Abstract

**Purpose:**

In geographic atrophy (GA), choroidal vessels typically appear on structural optical coherence tomography (OCT) as hyperreflective round areas with highly reflective borders. We observed that some GA eyes show choroidal round hyporeflectivities with highly reflective borders beneath the atrophy, and futher investigated the charcteristcs by comparing structural OCT, indocyanine green angiography (ICGA) and OCT angiography (OCT-A).

**Methods:**

Round hyporeflectivities were individuated from a pool of patients with GA secondary to non-neovascular age-related macular degeneration consecutively presenting between October 2015 and March 2016 at the Medical Retina & Imaging Unit of the University Vita-Salute San Raffaele. Patients underwent a complete ophthalmologic examination including ICGA, structural OCT and OCT-A. The correspondence between choroidal round hyporeflectivities beneath GA on structural OCT and ICGA and OCT-A imaging were analyzed.

**Results:**

Fifty eyes of 26 consecutive patients (17 females and 9 males; mean age 76.8±6.2 years) with GA were included. Twenty-nine round hyporeflectivities have been found by OCT in choroidal layers in 21 eyes of 21 patients (42.0%; estimated prevalence of 57.7%). All 29 round hyporeflectivities showed constantly a hyperreflective border and a backscattering on structural OCT, and appeared as hypofluorescent in late phase ICGA and as dark foci with non detectable flow in the choroidal segmentation of OCT-A. Interestingly, the GA area was greater in eyes with compared to eyes without round hyporeflectivities (9.30±5.74 and 5.57±4.48mm^2^, respectively; p = 0.01).

**Conclusions:**

Our results suggest that most round hyporeflectivities beneath GA may represent non-perfused or hypo-perfused choroidal vessels with non-detectable flow.

## Introduction

Geographic atrophy (GA) consists in the late stage of dry age-related macular degeneration (AMD). It is less common than neovascular AMD, but in individuals over 85 years of age the incidence of GA is approximately 4 times than exudative type [1].

GA is usually defined as a well-circumscribed, large (diameter > 200 μm), round or oval area of hypopigmentation in which choroidal vessels are more easily visualized because of the degeneration of the retinal pigment epithelium (RPE), followed by dysfunction and death of photoreceptors and choriocapillaris atrophy [2–4]. These morphological changes initially appear in the extrafoveal region and then advance into the fovea as the disease progresses, leading to severe vision loss [5].

Accurate clinical imaging techniques not only are necessary to monitor and predict GA progression but are also helpful in determining the endpoints for current ongoing clinical trials to evaluate therapies for GA.

Spectral domain optical coherence tomography (SD-OCT) is a rapidly evolving technology to investigate retinal and choroidal changes in various retinal degenerative diseases including GA [6–8]. Its role in imaging of GA has evolved over the last few years allowing to detect a large spectrum of morphological alterations. SD-OCT scans of GA show thinning of hyperreflective external band, corresponding to attenuation of RPE/Bruch’s complex, deeper hyperreflectivity due to the loss of outer layers including photoreceptors, and choroidal thinning that involves all vascular layers [9], mostly evident in a specific subtype of GA identified by fundus autofluorescence (FAF) imaging (i.e. “diffuse trickling”) [10]. Additional interesting changes visible on SD-OCT in eyes with GA are outer retinal tubulations (ORTs) caused by a degeneration of photoreceptors arranged in a circular or ovoid fashion [11], pseudocysts, seen as a hyporeflective circular shaped lesion and different from intra/subretinal fluid secondary to choroidal neovascularization (CNV) [12] and wedge-shaped subretinal hyporeflectivities delimited internally by the hyperreflective outer plexiform layer and externally by the hyperreflective Bruch’s membrane [13]. Moreover specific findings have been found also in the choroid by means of SD-OCT. Recently, Querques et al. [14] have described angular hyporeflective cavities with hyperreflective borders, called choroidal caverns, possibly derived from ghost vessels, in the choroid of some eyes with GA.

Optical coherence tomography angiography (OCT-A) is a new approach for visualizing retinal and choroidal vessels by detecting motion contrast from flowing blood. It has been applied in several retinal diseases, including dry AMD where it shows loss of choriocapillaris flow under the area of GA and asymmetric alterations at the margin of GA [15].

Using SD-OCT we observed that some GA eyes show choroidal round hyporeflectivities with highly reflective borders beneath the atrophy on structural B-scan. The aim of this study is to describe this novel SD-OCT finding, and to investigate the characteristics by comparing structural OCT B-scan, indocyanine green angiography (ICGA) and OCT-A, in order to provide hypothesis of its nature. Moreover we estimated the prevalence and evaluated its possible role in the evolution of the disease.

## Material and Methods

We reviewed the charts of consecutive patients with diagnosis of GA secondary to non-neovascular AMD that presented at the Medical Retina & Imaging Unit of the Department of Ophthalmology, University Vita-Salute, Ospedale San Raffaele in Milan between October 2015 and March 2016. This study adhered to the tenets of the Declaration of Helsinki. The San Saffaele Hospital ethics committee approved this study and all patients signed a written general consent to participate.

The inclusion criteria were: 1) age greater than 55 years, 2) diagnosis of atrophic AMD with GA (GA was defined as any sharply demarcated unifocal or multifocal area of absence of the RPE with visible choroidal vessels larger than 750 μm in diameter). The exclusion criteria included: 1) signs of CNV, including intraretinal or subretinal fluid, hemorrhage, subretinal fibrosis, 2) presence of any other retinal disorder potentially confounding the clinical assessment (e.g., diabetic retinopathy, retinal vein occlusion, retinal artery occlusion), 3) myopia greater than 6 diopters, 4) any previous treatment (e.g., laser photocoagulation, photodynamic therapy, intravitreal injections of anti-VEGF or steroids) 5) presence of significant media opacities (e.g., cataract or corneal opacity).

As part of standard clinical assessment, all patients underwent a complete ophthalmologic examination, including best-corrected visual acuity (BCVA) using Early Treatment Diabetic Retinopathy Study (ETDRS) charts, slit-lamp biomicroscopy, lens status examination, tonometry, indirect fundus ophthalmoscopy, infrared reflectance (IR), FAF, SD-OCT, fluorescein angiography (FA), ICGA and OCT-A.

Optical coherence tomography examinations were performed using Spectralis (Heidelberg Engineering, Heidelberg, Germany). Spectralis OCT uses SD technology with an 870-nm wavelength superluminescent diode, performs 40,000 A-scans per seconds with a scan depth of 1.8 mm, optical depth resolution of 7 μm and lateral optical resolution of 14 μm. The Spectralis software allows averaging a variable number of single images in real time (ART [Automatic Real Time] Module; Heidelberg Engineering) by means of automated eye tracking and image alignment based on cSLO images. The SD-OCT minimum acquisition protocol included 241 horizontal linear B-scans, each composed of 16 averaged OCT B-scans (768 A-scans per line) at 30 μm intervals, covering an area of 30 degrees by 25 degrees (approximately 8.5 x 7 mm^2^ area). To achieve good visualization of the choroid, enhanced depth-imaging (EDI) OCT was used in all acquisitions.

OCT-A examinations were performed using AngioPlex OCT angiographic imaging installed on the High Definition (HD) OCT CIRRUS™ Model 5000 (Carl Zeiss Meditec, Dublin, CA). This OCT has an A-scan rate of 68.000 A-scans per second using a superluminescent diode with a center wavelength of 840 nm and a bandwidth of 90 nm. To evaluate the morphology we used a 3 x 3 mm scanning area, which is composed by 245 A-scans in each B-scan along the horizontal direction and 245 B-scan positions along the vertical direction. Each B-scan is repeated four times in the 3 x 3 mm area. Flow images are generated by an algorithm known as OCT microangiography-complex (OMAGC), which incorporates differences in both the phase and intensity information contained within sequential B-scans. Angioplex also incorporates FastTrac™ retinal-tracking technology to reduce motion artifacts.

In order to ensure a correct visualization and assessment of the choriocapillaris layer and of the choroidal layers we used the automatic segmentation provided by the system software with minor manual adjustments. A section from 60 to 90 μm below the RPE was used for visualizing the Sattler layer and from 90 to 120 μm below the RPE for the Haller layer.

Two trained examiners (EC and RS) analyzed the number of round hyporeflectivities and their correspondence on structural OCT B-scan, ICGA and OCT-A imaging. The same examiners also measured manually the atrophic areas on FAF images, the foveal involvement of the atrophic lesion, the localization of round hyporeflectivities in relation to the fovea (classified as subfoveal if they were <200 μm from the center or extrafoveal if they were >200 μm from the center) and to the atrophic areas (defined as under the edge of the GA or under the center), the greatest linear dimensions and area of round hyporeflectivities, and the choroidal thickness at the fovea and at the regions including round hyporeflectivities. The choroidal thickness at SD-OCT was defined as the distance between the RPE-Bruch’s membrane complex and the chorioscleral border.

Statistical analyses were performed using SPSS Statistics Version 20 (IBM, Armonk, New York, USA). Results of descriptive analyses are expressed as means±standard deviations for quantitative variables and as counts and percentages for categorical variables. Fisher’s Exact test was used to compare binary variables. Interobserver reproducibility was evaluated with intraclass correlation coefficients (ICC; 95% confidence intervals [CI]). The Gaussian distribution of continuous variables was verified with the Kolmogorov-Smirnov test. Comparisons of mean BCVA, macular choroidal thickness and atrophic area between eyes with and without choroidal round hyporeflective were performed using the Student’s t-test. In all analyses, p values <0.05 were considered as statistically significant.

## Results

Fifty eyes of 26 consecutive patients (17 females, 9 males) met the inclusion criteria and were included for the analysis. The mean age was 76.8±6.2 years (median, 77; range, 66–87 years). Twenty-four patients had GA in both eyes. One fellow eye was excluded for the presence of CNV and one fellow eye for the absence of GA. GA mean area was 7.14±5.33 mm^2^ (range, 0.70–22.77 mm^2^) and the foveal area was involved in 32 of 50 eyes (64%). Table 1 shows demographics and main clinical findings of the patients included.

**Table 1:** Demographics and main clinical features of the study population affected with geographic atrophy.

		Total Eyes	Eyes with round hyporeflectivities	Eyes without round hyporeflectivities	P value
**N**		50	21	29	
**Sex, n**		26	15	11	
	Male	9	4	5	0.449*
	Female	17	11	6	
**Age, years (mean ± SD)**		76.8±6.2	78.4±6.8	74.4±4.6	0.1†
**BCVA, LogMAR (mean ± SD)**		0.40±0.29	0.43±0.29	0.38±0.29	0.5†
**Subfoveal ChT, μm (mean ± SD)**		165.72±84.24	188.62±67.93	149.14±91.90	0.1†
**GA foveal involvement, n (percentage)**		32 (64%)	17 (81%)	15 (52%)	0.04*
**GA extension, mm^2^ (mean ± SD)**		7.14±5.33	9.30±5.74	5.57±4.48	0.01†

N: number; SD: standard deviation; BCVA: best-corrected visual acuity; ChT: choroidal thickness; GA: geographic atrophy

*: Fisher’s Exact test

†: Student’s t-test.

Among the 50 eyes analyzed, 21 eyes of 15 patients presented round hyporeflectivities on structural OCT B-scans (a total of 29 [mean 1.38 per eye] round hyporeflectivities). This accounts for 57.7% (38.7–76.7, 95% CI) estimated prevalence (per patient) of round hyporeflectivities in patients affected with GA secondary to atrophic AMD. Nine round hyporeflectivities were located in the Haller layer, 10 in the Sattler layer and 10 in both Sattler and Haller layers. Three areas were located at the edge of GA and 26 in the area of GA. Nine round hyporeflectivities were located subfoveal and 20 extrafoveal (Table 2).

**Table 2:** Choroidal round hyporeflectivities features on optical coherence tomography (OCT).

		Eyes with round hyporeflectivities
**Round hyporeflectivities on OCT, n (mean ± SD)**		1.38 ± 0.80
**Round hyporeflectivities localization**		
	Edge of GA	3
	Inside GA	10
**Round hyporeflectivities localization**		
	Subfoveal	9
	Extrafoveal	20
**Round hyporeflectivities localization**		
	Haller layer	9
	Sattler layer	10
	Haller and Sattler layers	10
**Round hyporeflectivities maximum diameter, μm (mean ± SD)**		67.7 ± 29.0
**Round hyporeflectivities area, μm^2^ (mean ± SD)**		4247 ± 3392
**ChT at the round hyporeflectivities site, μm (mean ± SD)**		171 ± 59

OCT: optical coherence tomography; n: number; SD: standard deviation; GA: geographic atrophy; ChT: choroidal thickness.

**Fig 1:**
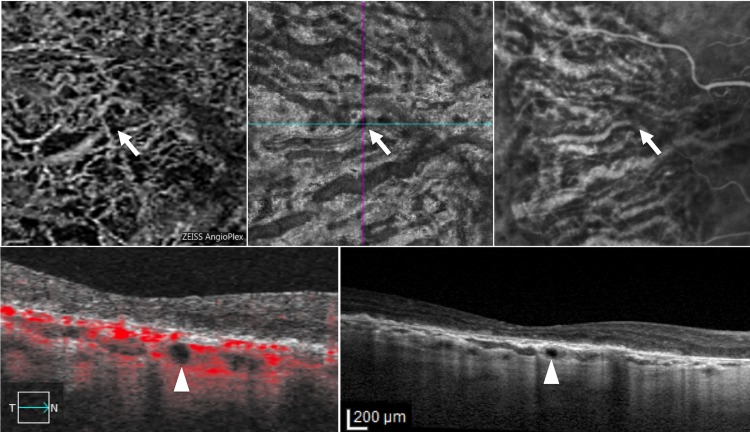
Optical coherence tomography angiography (OCT-A), spectral domain optical coherence tomography (SD-OCT) and indocyanine green angiography (ICGA) in the right eye of a patient with hyporeflective round areas. Choroidal segmentation on 3x3 OCT-A (first row, left panel) / en face imaging (first row, middle panel), and ICGA (first row, right panel) reveal, respectively, non detectable flow / focal hyporeflectivity and hypofluorescence (arrowhead) along the course of a large choroidal vessel. Structural SD-OCT B-scan with (second row, left panel) and without flow analysis (second row, right panel) discloses a round hyporeflectivity (arrowhead) localized in the large choroidal vessel layer within geographic atrophy, beneath the fovea.

**Fig 2:**
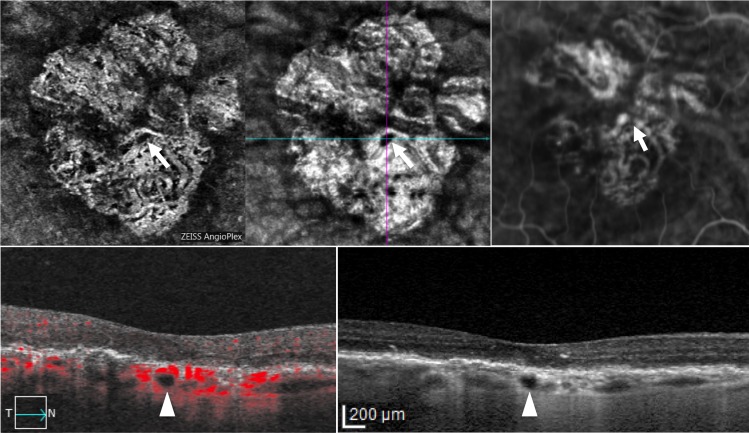
Optical coherence tomography angiography (OCT-A), spectral domain optical coherence tomography (SD-OCT) and indocyanine green angiography (ICGA) in the right eye of a patient with hyporeflective round areas. Choroidal segmentation on 3x3 OCT-A (first row, left panel) / en face imaging (first row, middle panel), and ICGA (first row, right panel) reveal, respectively, non detectable flow / focal hyporeflectivity and hypofluorescence (arrowhead) along the course of a large choroidal vessel. Structural SD-OCT B-scan with (second row, left panel) and without flow analysis (second row, right panel) discloses a round hyporeflectivity (arrowhead) localized in the large choroidal vessel layer within geographic atrophy, beneath the fovea.

On OCT B-scan, all choroidal hyporeflectivities were typically empty, round / oval, characterized by a hyperreflective border, and in most cases, by backscattering (Figs 1 and 2). Only 2 out of 29 hyporeflectivities did not show backscattering because they were overlaid by subretinal/sub-RPE hyperreflective material. The mean greatest linear dimension of round hyporeflectivities was 67.7±29.0 μm (range, 26–134 μm; ICC = 0.994 [0.987–0.997]). The mean round hyporeflectivities was 4247±3392 μm^2^ (range, 530–14102 μm^2^; ICC = 0.991 [0.980–0.996]). Mean choroidal thickness at the round hyporeflectivities site was 171±59 μm (range, 71–280 μm; ICC = 0.998 [0.995–0.999]), and 165±84 μm (range, 45–412 μm; ICC = 0.997 [0.994–0.998]) in subfoveal area. Interobserver variability (ICC; 95% CI) was excellent for all these measurements.

**Fig 3:**
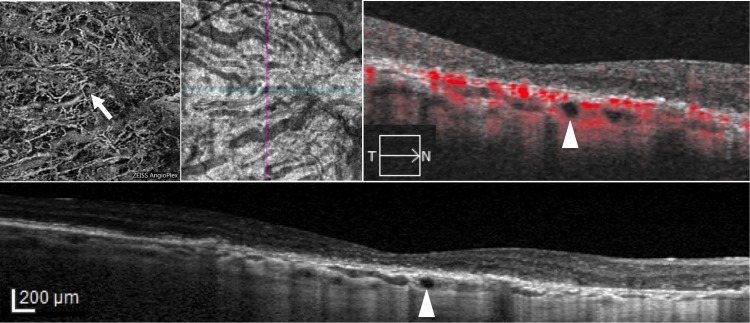
Optical coherence tomography angiography (OCT-A) and spectral domain optical coherence tomography (SD-OCT) at 6 months follow-up visit in the right eye of a patient (previous examination reported in Fig 1) with hyporeflective round areas. Choroidal segmentation on 3x3 OCT-A (first row, first panel) / en face imaging (first row, second panel), structural SD-OCT B-scan with (first row, third panel) and without flow analysis (second row, first panel) reveal no changes in size and shape six months after hyporeflective round areas identification.

**Fig 4:**
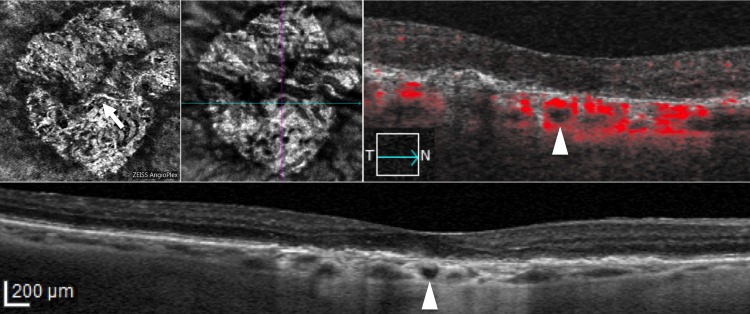
Optical coherence tomography angiography (OCT-A) and spectral domain optical coherence tomography (SD-OCT) at 6 months follow-up visit in the right eye of a patient (previous examination reported in in Fig 2) with hyporeflective round areas. Choroidal segmentation on 3x3 OCT-A (first row, first panel) / en face imaging (first row, second panel), structural SD-OCT B-scan with (first row, third panel) and without flow analysis (second row, first panel) reveal no changes in size and shape six months after hyporeflective round areas identification.

In all cases, the round hyporeflectivities were clearly hypofluorescent in late phase ICGA (Figs 1 and 2), and all 29 areas (21 eyes) appeared as dark foci with non detectable flow in the choroidal segmentation of OCT-A (Figs 1 and 2). Interestingly, for some hyporeflectivities (8 in 7 out of 21 eyes) it was possible to distinguish a hyporeflective sinusoidal tract in the sclera. Among the 21 eyes presenting round hyporeflectivities, structural OCT B-scans and OCT-A follow-up was available in 18 eyes (86%) of 13 patients. Mean follow-up was 5.8±1.7 months (median, 6; range, 2–8 months). Interestingly, all choroidal round lesions did not change in size, shape and anatomical location as shown in Figs 3 and 4.

The atrophic area was greater in eyes with compared to eyes without round hyporeflectivities (9.30±5.74 and 5.57±4.48 mm^2^, respectively; p = 0.01) and involved more often the fovea (17 out of 21 eyes [81%] and 15 out of 29 eyes [52%], respectively; p = 0.04). No statistical differences were found in 21 eyes with compared to 29 eyes without round hyporeflectivities with respect to BCVA (0.43±0.29 and 0.38±0.29 LogMAR, respectively; p = 0.5) and subfoveal choroidal thickness (188±67 μm and 149±91 μm, respectively; p = 0.1).

## Discussion

In this study, we described hyporeflectivities observed in the choroid of eyes affected by GA as a new OCT finding. They have been found in 42% of eyes (21 out of 50, mean 1.38 per eye) and appeared on structural OCT B-scan as round / oval hyporeflectivities, typically delimited by hyperreflective border characterized by backscattering, and localized both in Haller and in Sattler layers (Figs 1 and 2). They had diameters comparable to choroidal vessels [16–19] (67.7±29.0 μm in our series), from which choroidal round hyporeflectivities were easily distinguished. In fact, choroidal vessels beneath GA, typically appear as slightly hyperreflective, because of the presence of blood (regularly flowing inside), with highly reflective borders representing the vessel wall in a thinner choroid. Moreover, they appear as particularly reflective compared to choroidal vessels in areas not affected by GA because of higher penetration of OCT wavelength due to absence of RPE.

To the best of our knowledge, choroidal round hyporeflectivities in patients with GA have never been described previously in literature. On the other hand, the thinning of the choroid involving all vascular layers (in our series the mean subfoveal choroidal thickness was 165±84 μm) has been constantly reported in different studies [9–14, 20].

The choroid is the vascular layer of the eye and may be divided into three layers. From retina to sclera, the choroid comprises the choriocapillary, defined as the capillary layer consisting of a network of wide-bore capillaries with saclike dilatations, lined by a continuous layer of fenestrated endothelial cells, and the vessel layer consisting of loose connective tissue in which are embedded numerous medium (Sattler’s layer) and large-sized (Haller’s layer) blood vessels. The principal function of the choroid is to nourish the RPE with its blood vessels and the outer layers of the retina; thus, a morphological and/or functional alteration of the choroid may lead to retinal photoreceptor dysfunction and death.

Until recently, the choroid was evaluated in vitro by histological analysis, while in vivo the main tool for visualization of choroidal vessels and circulation under the RPE was ICGA. However, it is an invasive technique based on dye injection and does not enable three-dimensional evaluation of the choroid and acquisition of data on choroidal thickness. OCT is able to overcome these limitations, by providing cross-sectional images of the anatomy of choroidal layers. In particular, EDI OCT is a technology that uses low signal strength and low resolution but increased depth over conventional SD-OCT to acquire detailed cross-sectional images of the choroid [21]. By means of OCT several findings have been recently described in the retina of GA patients [11–13]. However, only a peculiar OCT finding has been described in the choroid of some GA areas of atrophic AMD eyes, the choroidal caverns [14]. One could argue that the finding here reported may be confused for choroidal caverns. However there are several differences between the two findings which include shape, borders and anatomical locations. Choroidal caverns appear on OCT as gaping hyporeflective cavities in the choroid, typically angular without hypereflective borders often with punctate/linear hypereflectivities internally; whereas choroidal round hyporeflectivities are round / oval hyporeflectivities, typically delimited by hypereflective border characterized by backscattering. We hypothesize that the round hyporeflectivities described in the current study could represent precursors of choroidal caverns recently reported by Querques et al. [14], and, indeed, one could argue that some similitudes could be highlighted in certain cases between these two findings (as displayed in Fig 4, reference 14). Although alternative explanations cannot be ruled out, they could represent non-perfused or hypo-perfused vessels actually deriving from vessels sclerosis process. This hypothesis is supported from their ICGA and OCT-A appearance. On ICGA choroidal round hyporeflectivities constantly corresponded to hypofluorescent areas, and on OCT-A they were characterized by non detectable flow. OCT-A is a novel tool based on the concept that in a static eye the only moving structure is blood flowing through vessels; however, this technology is influenced by sensitivity threshold. Therefore areas of non detectable flow do not mean that they are without flow, as they could correspond to areas characterized by flow slower or higher than the detection range of OCT-A [22]. This implies that choroidal round hyporeflectivities may also represent areas of choroidal vessels that, despite anatomically normal, are characterized by irregular blood flow. Such irregular blood flow could be due to different reasons, including a tortuous course either within the choroid, or from the retrobulbar space to the choroid. It is noteworthy that in 8 choroidal round hyporeflectivities it was possible to distinguish a hyporeflective sinusoidal tract in the sclera, suggesting a continuum with scleral perforating vessels (typically appearing as hyporeflective) [23].

A different origin for the choroidal round hyporeflectivities could be hypothesized. They could represent lipid globules, derived by fatty degeneration. Lipid deposits in the form of globules have been described firstly in 1966 by Friedman [24] in the choroid of a number of normal eyes post mortem. They have been found typically in cluster at the posterior pole in all layers of the choroid, in the majority of cases near the Bruch’s membrane, but not associated with significant ocular pathology. Recently, Glover et al. [25] described lipid globules as similar to choroidal vessels in diameter, on histological examination of eyes with and without AMD. The authors found these globules in 4 out of 13 eyes with GA and proposed that they may be fat emboli, products derived from red blood cell membrane breakdown or consequence of increased extracellular lipase activity.

Another possibility could be that choroidal round hyporeflectivies are large single cells with a relatively uniform internal composition. In particular, tissue macrophages may reach 60–80 μm in diameter and may become laden with lipid droplets that appear hyporeflective by OCT [26].

Interestingly, in our series, patients with compared to patients without choroidal round hyporeflectivities showed a larger mean atrophic area. This could be due to a major impairment of choroidal circulation, which is in agreement with both hypothesis of non-perfused or hypo-perfused vessels and lipid globules for the origin of choroidal round hyporeflectivities. Given that we found choroidal round hyporeflectivities to be associated with larger GA area, we suggest a possible prognostic value for this previously unreported finding.

Our study has several limitations mainly due to retrospective analysis, the small number of included eyes, and the absence of follow-up examination. Histologic analysis is warranted to confirm our findings and the proposed hypothesis for their origin.

In conclusion, we describe a new OCT finding in patients affected by GA secondary to atrophic AMD. Our results suggest that choroidal round hyporeflectivities beneath GA may represent hypo- or non-perfused choroidal vessels; alternatively, they may represents lipid globules within the choroids. Future studies investigating the progression are warranted to better understand the pathogenesis and the possible prognostic value of this previously unreported finding.
